# Teaching antenatal hand expression: a feasibility study in an inner urban U.S. hospital

**DOI:** 10.1186/s13006-023-00578-w

**Published:** 2023-08-10

**Authors:** Sally Chen, Yukiko Washio, Angela Liu, Colette Acker, Gail Herrine

**Affiliations:** 1 Obstetrics, Gynecology, and Reproductive Sciences, Temple University Hospital, Philadelphia, PA USA; 2 RTI International, Research Triangle Park, NC USA; 3 Breastfeeding Resource Center, Abington, PA USA

**Keywords:** Breastfeeding, Prenatal hand expression, Antenatal hand expression, Barriers to breastfeeding, Colostrum expression, Antenatal milk expression, Pregnancy

## Abstract

**Background:**

Many women have low confidence in breastfeeding and have concerns regarding low milk volume or discomfort with breastfeeding. Antenatal hand expression may be an opportunity to help women feel more comfortable with breastfeeding and help promote exclusive breastfeeding. A study at a hospital in Philadelphia, Pennsylvania, U.S. assessed the feasibility of teaching antenatal hand expression at 39 weeks among socioeconomically disadvantaged populations, overall participant satisfaction and adoption of hand expression and breastfeeding.

**Methods:**

From March 2020 to June 2021, women recruited at 34–39 weeks were taught to hand express, collect, and store colostrum. Starting from 39 weeks, participants were asked to practice hand expression 1–3 times / day until delivery, log their experiences, and store colostrum expressed. Women were contacted to encourage continued hand expression and answer any questions. Postpartum, a survey assessed satisfaction with hand expression and issues encountered. The survey also inquired about breastfeeding plans and barriers, and whether women were exclusively breastfeeding (defined as infants who received only breastmilk from the time of birth). Chart review of postpartum or well-baby visit notes determined whether women continued breastfeeding.

**Results:**

Of the 29 participants, 72% (21/29) reported hand expressing at home, and no women reported contractions when hand expressing. Participants rated mean satisfaction of 8.1/10 (SD = 1.62) with antenatal hand expression, mean satisfaction of 9.4/10 (SD = 0.90) toward hand expression education, mean likelihood of 9.4/10 (SD = 1.24) recommending hand expression to others, and a mean score of 8.1/10 (SD = 1.69) on how helpful hand expression was in breastfeeding initiation. 90% (26/29) of women initiated breastfeeding after birth and 72% (21/29) exclusively breastfed on discharge, but only 11/29 (38%) continued exclusively breastfeeding when re-assessed 4–6 weeks postpartum. Barriers included maternal discomfort, low milk supply, and maternal or infant illness.

**Conclusions:**

This study suggests that women in an urban setting would be willing to practice antenatal hand expression. A larger and adequately powered study could be feasible to determine associations between antenatal hand expression and breastfeeding rates and confidence.

**Supplementary Information:**

The online version contains supplementary material available at 10.1186/s13006-023-00578-w.

## Background

Antenatal hand expression of breastmilk has started to become a strategy to prepare the pregnant mother for breastfeeding and has been utilized as a tool to hopefully improve maternal breastfeeding confidence and breastfeeding outcomes. Previous qualitative studies have showed that mothers reported increased confidence in breastfeeding if they had experiences of antenatal milk expression [1–4] and that milk stored from antenatal milk expression provided women with a sense of security [2–4]. A cross-sectional study of 688 women also showed that 80.9% of surveyed women would consider antenatal breastmilk expression if it was found to be helpful to prepare for breastfeeding [5]. Another retrospective cohort study involving antenatal hand expression in women with diabetes found that infants born to mothers who hand expressed antenatally were less likely to receive formula during hospital admission [3], suggesting that antenatal hand expression may position women with diabetes to successfully breastfeed. However, these studies also reported similar negative emotions and feedback toward hand expression, including pain and discomfort [1, 2, 4]; embarrassment [1, 4]; fears of inducing premature labor [2] or causing harm to their baby [3]; disappointment if low volume of milk was expressed [2, 3]; or futility if their breastmilk was not used [3, 4].

Interest regarding the safety and efficacy of antenatal hand expression as an effective technique for obtaining colostrum has also been increasing [6]. Many initial studies in the literature had implemented antenatal hand expression in women with diabetes, as infants born to women with diabetes are at higher risk of infant hypoglycemia and collecting and storing colostrum can potentially treat or prevent infant hypoglycemia [7, 8]. One study found that women with diabetes who hand expressed had delivered about one week earlier than their control group counterparts [7] and higher neonatal special care unit admissions were noted in two similar studies [7, 8]. Yet another study reported no participants had any uterine contractions related to antenatal breastmilk expression [9]. All the studies were small-scale and underpowered to detect true effects. Currently, the only larger-scale study, the Diabetes and Antenatal Milk Expression (DAME) trial, established no difference in maternal complications, such as in labor onset, type of delivery, blood loss, or maternal hypoglycemia, as well as no difference in neonatal outcomes, including neonatal intensive care unit admissions, shorter mean gestation ages, lower birth weight, or lower Apgar scores, among low-risk women with diabetes who began hand expression starting at 36 weeks [10]. More recently, in a pilot feasibility study of a structured antenatal breastmilk expression intervention among nulliparous women who started hand expression at 37 weeks’ gestation, no participants reported symptoms of decreased fetal movement, prolonged uterine tightening, or vaginal bleeding during or directly following antenatal hand expression [11], but further research is needed to validate these results.

Previous studies have also examined attitudes toward antenatal education of hand expression techniques through infographics [12] or video instruction [13] and found that both were acceptable ways to provide instruction about antenatal hand expression and were well-received by study participants. Prenatal breastfeeding education is a recommended strategy from the United States Preventive Services Task Force and the WHO / UNICEF to increase breastfeeding rates [14]. Among current literature related to breastfeeding, some studies show that prenatal breastfeeding interventions led to higher self-efficacy scores and greater breastfeeding rates [15–17] while other meta-analyses show that breastfeeding education and counseling has no impact on breastfeeding initiation or exclusive breastfeeding rates [18, 19]. Still, among low-income mothers who had Medicaid insurance or were uninsured, participants agreed that educational breastfeeding interventions would have helped them exclusively breastfeed [20]. In addition, results of the DAME trial suggested that antenatal hand expression was moderately associated with exclusive breastfeeding of newborns in the first twenty-four hours of life and during the initial hospital stay [10], but further studies would be needed to evaluate how antenatal breastmilk expression could increase long-term breastfeeding rates.

The aim of this study was to evaluate the feasibility and acceptability of informational material and individual prenatal consultation to women’s willingness to perform antenatal hand expression. Given limited data on safety of prenatal hand expression, our institutional review board approved starting our prenatal hand expression intervention at 39 gestational weeks, when elective inductions of labor are also routinely offered [21]. Thus, the study was able to evaluate the feasibility of education and consultation on antenatal hand milk expression starting at 39 gestational weeks in breastfeeding initiation and continuation among the low-income population.

## Methods

This single-institution study was reviewed and approved by the institutional review board at Temple University in Philadelphia, Pennsylvania. All eligible women who were planning on breastfeeding their infants were approached during routine prenatal care by the study clinician (GH) between 34 and 39 weeks’ gestation and given information regarding the study from March 2020 to June 2021. The study was described in detail, including discussing the educational session, collecting breastmilk, freezing it and bringing it into the hospital when giving birth. Written informed consent was obtained from study participants. The participants were given the opportunity to take the blank consent form home with them to review and discuss further at following visits. The sample size of 30 was a convenience sample based on the study timeline of one year but was considered adequate to explore feasibility issues. No compensation was offered to study participants.

The study inclusion and exclusion criteria were: (1) a pregnant female; (2) aged 18 years or older; (3) receiving prenatal care at the study site; (4) able and willing to provide informed consent; (5) had no contraindications to breastfeeding. No other specific pregnancy complications excluded women from participating.

Initially, the plan for the study was to have the lactation consultant (CA) provide the education live and in the office setting, however the COVID-19 pandemic started just after institutional review board approval for the study was received and modifications needed to be made to the study protocol. The participants continued to be enrolled in the office during visits, but the educational part of study was conducted via Zoom sessions.

**Fig. 1:**
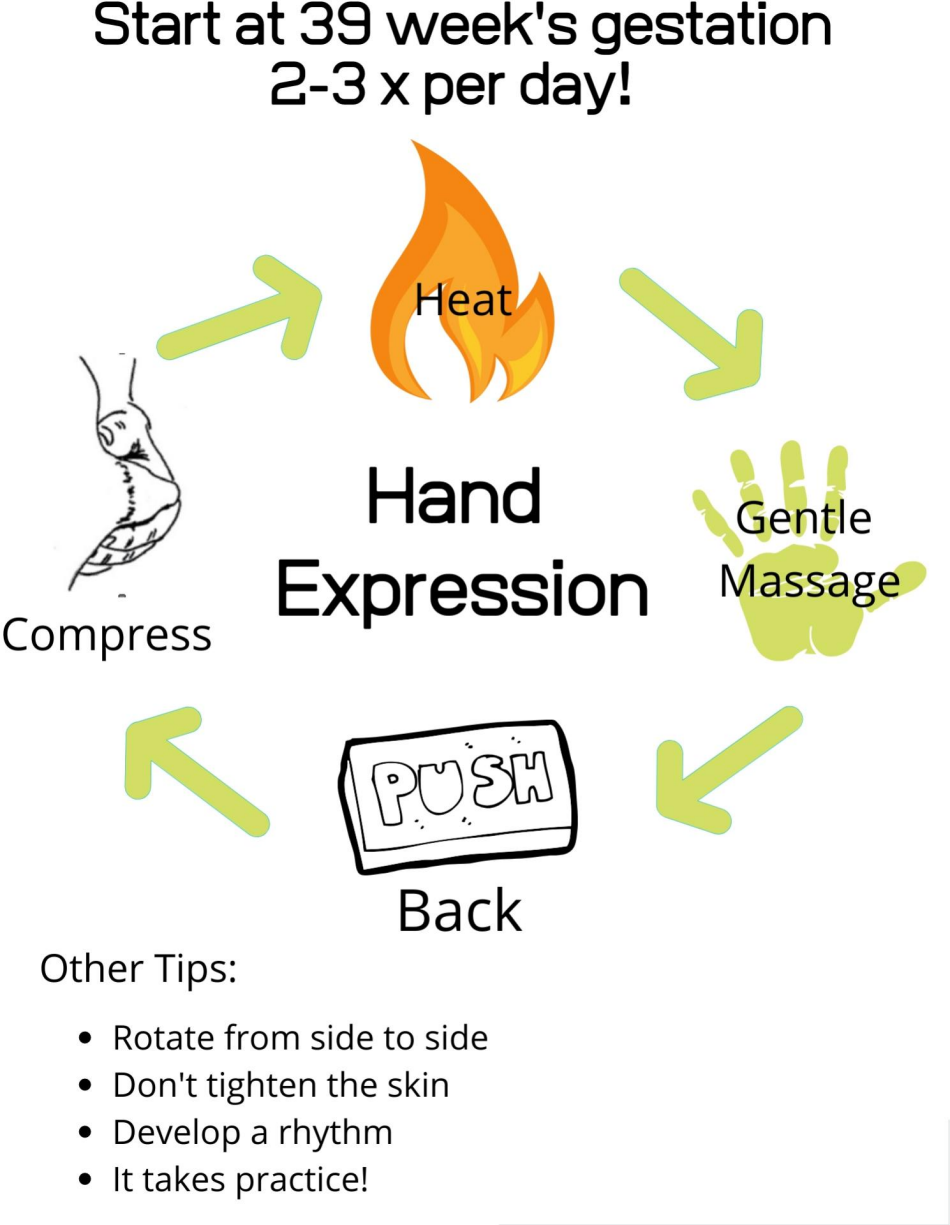
Infographic to encourage participants to practice hand expression starting at 39 weeks gestation

**Fig. 2:**
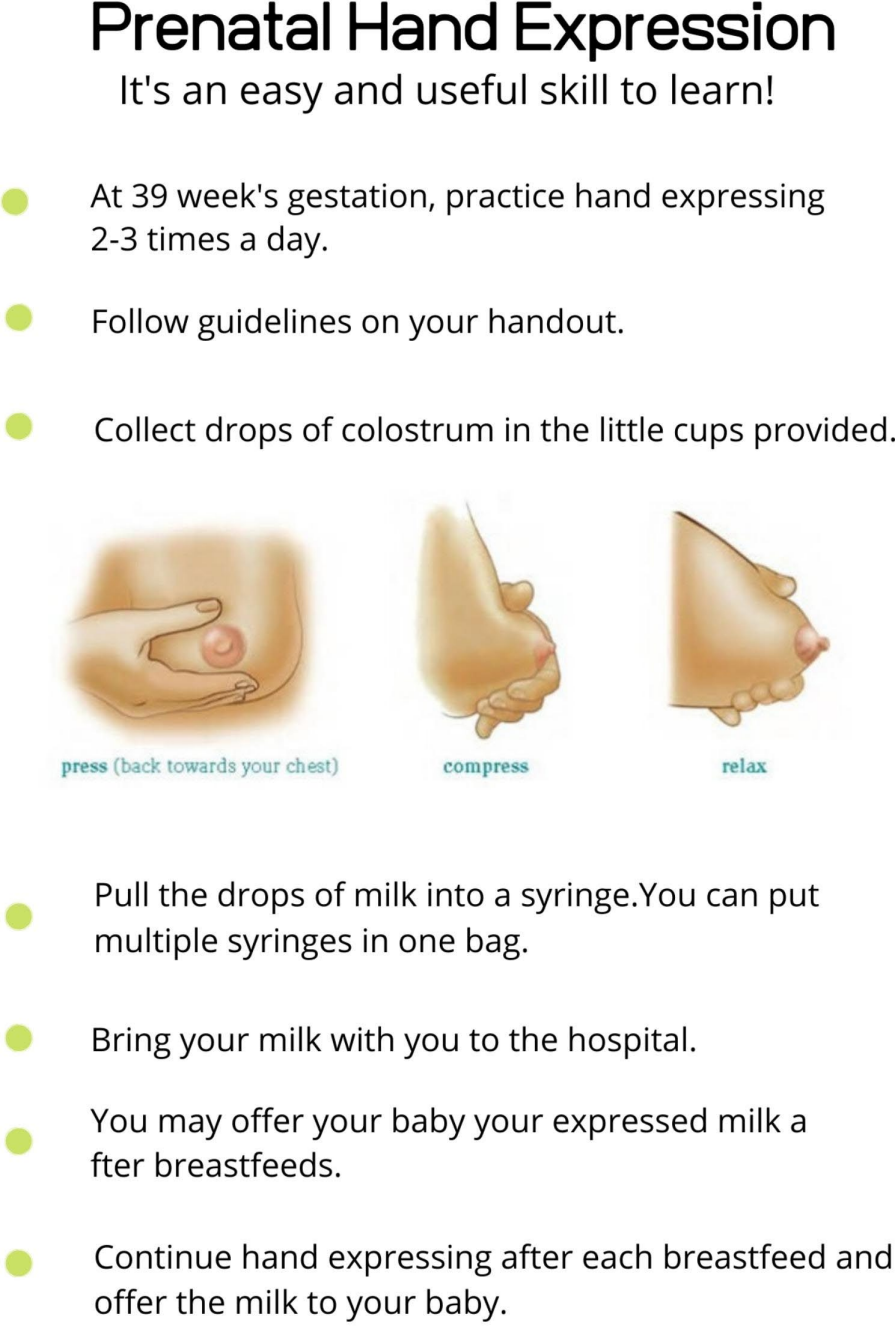
Instructions on how to hand express, collect, and store breastmilk

After consenting to take part in the study, participants were given a cooler, ice pack, milk collection cups, syringes, instruction sheets and a log spreadsheet (which included date and time of expression and if the participant experienced any contractions during hand expression). The lactation consultant contacted the participants and set up a call. The participants met with an International Board-Certified Lactation Consultant (IBCLC) via pre-arranged Zoom call, during which multimedia breastfeeding material was reviewed and the women were taught how to hand express colostrum, as part of her routine practice. Participants were also given handouts as seen in Figs. 1 and 2. A single lactation consultant provided education for all study participants. Furthermore, participants were instructed to begin hand expression at home at 39 weeks’ gestation one to three times daily; to record output; to log their discomfort or contractions; and to bring the frozen colostrum to the hospital in the cooler with the ice pack when they came to hospital for delivery. Participants were given a sample log sheet which encouraged them to record how many times they hand expressed; for how many minutes they hand expressed; how confident they felt with hand expression; and whether they felt contractions or discomfort (see Additional file 1). After the initial consultation, women also received two text message reminders a few days later as well as a phone call about four days to one week later, to help encourage continued practice of hand expression and answer any questions.

After delivery, the women completed a postpartum survey (see Additional file 2), which included several questions rated on a 10-point Likert scale. The postpartum survey was administered to the participants while still on the postpartum unit by resident physicians on the Obstetrics service in the Temple University Hospital Obstetrics and Gynecology residency program. On the survey, participants were asked how satisfied they were with hand expression overall; whether they had ever hand expressed before this study (in their prior pregnancies); whether they practiced hand expression at home and how many times; whether they encountered any problems and if so, what they were; whether they thought practicing hand expression helped initiate breastfeeding more easily; and whether they would recommend hand expression to family or friends.

To assess breastfeeding practices, the survey asked women whether they had initiated breastfeeding and if they were exclusively breastfeeding, which is defined as infants who received only breastmilk from the time of birth to discharge from the hospital, including no other foods or liquids including infant formula or water. Women were also asked if they had breastfed in the past in their previous pregnancies. Participants also rated their likelihood of continuing exclusively breastfeeding and what barriers they faced. The mean satisfaction and helpfulness scores and standard deviation (SD) for each of the categories stated above were calculated. Retrospective chart review of postpartum progress notes and discharge summaries after birth prior to hospital discharge determined whether women were exclusively breastfeeding, formula feeding only, or both. Well-baby visit notes or outpatient postpartum visit notes ranging from two to six weeks after discharge from the hospital estimated women’s breastfeeding practices several weeks postpartum.

Demographic data was extracted from the charts to include, age, gestational age recruited, parity, race, ethnicity, employment status and insurance type (Medicaid or private). Breastfeeding rates (exclusivity)was extracted from the mother / baby chart by reviewing every feed documented and if formula was ever given. (This was the same data the hospital collects for Joint Commission certification of a Baby-Friendly Hospital).

## Results

A total of 30 women gave consent for this study: 27 delivered at Temple University Hospital; three delivered at an outside hospital; and one was lost to follow up. Approximately half of the women, 47% (14 / 30), were 18–24 years old, 30% (9 / 30) were 30–35 years old, and 20% (6 / 30) were 25–29 years old. About 40% (12 / 30) of our study population were Hispanic, followed by 33% (10 / 30) identified as non-Hispanic white, and 20% (6 / 30) identified as non-Hispanic black. A majority, 80%, of the women received health insurance through Medicaid, and the remainder had private health insurance. No participants in our study sample were uninsured. In addition, 20% of them were employed. In this study population, 23% (7 / 30) of participants were enrolled in the study at 35–36 weeks’ gestation; 70% (21 / 30) of participants enrolled at 37–38 weeks; and 6% (2 / 30) of participants enroled at 39 weeks’ gestation. Mean gravidity of participants was 2.76 (SD = 2.04) and mean parity was 0.96 (SD = 1.21). The mean gestational age at delivery was 39.8 (SD = 0.52) weeks.

Of the 29 women who completed the postpartum survey shown in Additional File 2, overall, most were very satisfied with the practice of hand expression. Only 14% (4 / 29) of survey participants reported hand expression, antepartum or postpartum, in a previous pregnancy, but 72% (21 / 29) of the participants reported practicing and utilizing hand expression techniques at home in this study. Though participants were meant to document their hand expression experiences daily, including recording how many times they practiced each day and the amount of colostrum expressed, participants were inconsistent in their documentation, so this data was not accurately captured. However, from the 10 antenatal hand expression log sheets that were submitted, none of the women who initiated hand expression reported feeling contractions when practicing hand expression. Regarding overall satisfaction of hand expression, participants rated a mean score of 8.1 out of 10 (SD = 1.62). Women rated a mean score of 9.6 out of 10 (SD = 0.90) for satisfaction toward the hand expression education provided by the IBCLC and a mean score of 9.4 out of 10 (SD = 1.24) for likelihood of recommending hand expression to others. Most importantly, participants reported a mean score of 8.8 out of 10 (SD = 1.69) in how helpful hand expression was in initiating breastfeeding. However, 69% (9 / 13) of women reported having a “low output” of milk and 31% (4 / 13) reported “discomfort” in hand expression.

From data collected from our institution, a Baby-Friendly Hospital, 889 women delivered at our hospital during our study period. In this period, 12% (107 / 889) of women discharged from the hospital after birth were exclusively breastfeeding. In comparison, of the 29 participants in our study who completed the postpartum survey [see Additional File 2], 90% (26 / 29) had initiated breastfeeding in the hospital shortly after delivery, and 72% (21 / 29) were exclusively breastfeeding upon discharge from the hospital. 55% (16 / 29) of all the women in the study expressed interest in continuing to exclusively breastfeed. 69% (11 / 16) of the multiparous women had reported breastfeeding their older children. However, from reviewing well-baby and postpartum visit notes that ranged from two to six weeks postpartum, only 38% (11 / 29) were documented as still exclusively breastfeeding, 34% (10 / 29) were breastfeeding and supplementing with formula, and 28% (8 / 29) were exclusively using formula.

Many women reported multiple barriers to breastfeeding. The most common barrier was “low milk supply,” cited by 39% (9 / 23) of women, leading some mothers to supplement with formula and others to give up breastfeeding altogether. Other reasons for not exclusively breastfeeding including infant and maternal illness (22%; 5 / 23), poor latch (17%; 4 / 23), inconvenience (17%; 4 / 23), discomfort (9%; 2 / 23), and having to return to work (9%; 2 / 23).

## Discussion

This study examined the feasibility of antenatal hand expression at 39 gestational weeks in a socioeconomically disadvantaged population. With the implementation of our intervention, we found that 72% (21 / 29) of participants had initiated antenatal hand expression and were highly satisfied with the intervention. These women also felt antenatal hand expression was very helpful in initiating breastfeeding even though they started at 39 weeks meaning that most women only had a few days or up to a week until delivery to hand express breastmilk. Our feasibility study suggests that women in our low-income population were willing to accept antenatal hand expression and breastfeeding education, adopt hand expression techniques, and to initiate breastfeeding in the hospital. 90% of our study participants initiated breastfeeding in the hospital, and 72% were exclusively breastfeeding upon discharge. In our urban North Mid-Atlantic institution, we estimate generally only 12% of women are breastfed exclusively on discharge from the hospital, however, this percentage may be skewed as it may also include women who did not intend to breastfeed after delivery and more specific data were not readily available. Additionally, a larger and more adequately powered study would need to be performed to determine whether antenatal hand expression and prenatal education can help increase breastfeeding initiation and exclusivity rates in our population.

While some clinicians propose that antenatal hand expression could be an acceptable practice to promote breastfeeding [5, 22–25], more research and higher quality studies are needed to delineate optimal time of commencement, safety and impact on breastfeeding rates. In our study, women started hand expression later than in other studies [10, 11], and none of our study participants experienced any symptoms of premature uterine contractions, though this was only anecdotally reported. Study participants were not formally interviewed in a standardized fashion on their experiences. Future studies may consider starting hand expression earlier than 39 weeks to allow for more time to practice and build confidence in hand expression and increase self-perceived confidence with breastfeeding.

The finding of over 70% adoption of antenatal hand expression and overall satisfaction with our intervention among study participants may represent the importance of prenatal education and counseling. Though there is conflicting evidence on whether prenatal education directly impacts breastfeeding rates [18, 19], some studies suggest that additional educational or multi-media information are critical in underserved populations [15, 16, 20, 26], which are similar to our participant cohort, the majority of whom are unemployed and insured only through Medicaid. Some studies cite that women who did not receive prenatal education on breastfeeding had up to a 1.5–2.1 times higher risk of stopping exclusive breastfeeding [27, 28]. Individual meetings with an IBCLC and close follow-up from our research team as part of our study implementation could have been an important factor in our participants’ self-reported breastfeeding confidence and satisfaction, but larger and more extensive studies would have to be performed to assess whether these individualized meetings can impact rates of breastfeeding initiation and exclusive breastfeeding.

For those women in our study who hand expressed at home prior to birth, effort and participation was also largely self-motivated, and participants encountered several barriers to hand expression, with 69% of women who hand expressed in our study reporting “low milk supply” as their greatest barrier. In our study intervention, we did not discuss with participants what mean total volume of expected antenatal breastmilk expressed should be, which is estimated to only be about 5.5 mL [10, 11]. Additionally, we did not have participants record the volume they expressed, so it is unclear whether participants truly had low volumes of antenatal breastmilk expressed or whether participants had inaccurate expectations. It is possible that some women became frustrated after a few attempts at hand expression, for example, if they did not collect what they perceived to be enough breastmilk, or if their attempts were compounded by “discomfort” accompanying postnatal breastfeeding and they preferred the “convenience” of infant formula. Several women in our study chose to move away from exclusive breastfeeding and introduce infant formula instead. In the current literature, many of the reported barriers to antenatal hand expression and exclusive breastfeeding are similar to those cited by our study participants, including women having reported the perception of insufficient milk [28, 29], concerns about maternal or infant health, pain or discomfort, early in-hospital formula supplementation [30], or maternal return to school or work [31]. Future studies are warranted to further explore these barriers.

Some limitations in this study are acknowledged. The relatively small convenience sample size of 30 women, with 29 women having completed the postpartum survey, is the main limitation to this study. As a feasibility study, it may suggest that women in our population are open to antenatal hand expression education and lactation consultant education, but a larger study would be needed to assess whether antenatal hand expression impacted breastfeeding initiation and exclusivity rates. Also, our study results may not be generalizable to other populations dissimilar to ours, which is primarily an underserved, urban population in the United States. There is also a likelihood of potential sampling bias as women who voluntarily participated in our study may have been more highly motivated to participate in breastfeeding and hand expression than women who did not participate. Our study did not capture how many participants were approached, how many declined study enrolment and why. With the COVID-19 pandemic, there was a subsequent pause during recruitment period, requiring over a year to recruit our study sample, and modifications were made in the protocol to accommodate changes from in the office to remote operations of lactation consultations. As a result of COVID-19, hospital visitor policy changes, participants and visitors were not allowed frequent entry or exit of hospital, so participants were given an insulated bag with ice packs to transport collected colostrum, which may also have contributed to participant willingness to hand express, store, and transport their own colostrum.

## Conclusion

Results of this feasibility study suggest that women in urban, socioeconomically disadvantaged populations like our study population may be open and willing to practice antenatal hand expression. The practice of hand expression, as reported by our study participants, was met with high satisfaction scores, high self-reported likelihood of recommending to others, and high self-perceived helpfulness in initiating breastfeeding. Antenatal hand expression could be an important technique in increasing women’s comfort with their breasts and possibly impact breastfeeding initiation, continuation, and exclusivity rates, warranting future, larger, and fully-powered studies to examine the impact of antenatal hand expression on breastfeeding rates.

### Electronic supplementary material

Below is the link to the electronic supplementary material.

Additional file 1: Daily Hand Expression Tracker. Handouts provided to participants to record hand expression attempts, volume of expressed milk, any contractions, and discomforts when expressing

Additional file 2: Postpartum Survey. Assessment of participants in the hospital after delivery regarding satisfaction with hand expression and issues encountered, breastfeeding plans and barriers, and exclusive breastfeeding practices

## Data Availability

The data that support the findings of this study are available on request from the corresponding author [SC]. The data are not publicly available due to them containing information that could compromise research participant privacy / consent.

## Abbreviations

IBCLC: International Board-Certified Lactation Consultant
SD: standard deviation
WHO: World Health Organization
