# Supplementary material for: Teaching antenatal hand expression: a feasibility study in an inner urban U.S. hospital
Source: Int Breastfeed J. 2023 Aug 10;18:39. doi: 10.1186/s13006-023-00578-w (PMC10416364; doi:10.1186/s13006-023-00578-w)
Supplement: Supplementary file 1 — Additional file 1: Daily Hand Expression Tracker. Handouts provided to participants to record hand expression attempts, volume of expressed milk, any contractions, and discomforts when expressing [file 13006_2023_578_MOESM1_ESM.pdf]

Additional File 1: Daily Hand Expression Tracker

Daily Hand Expression Tracker

Today's Date ___________________

How many times did you try to hand express today?

☐ 0 ☐ 1 ☐ 2 ☐ 3 ☐ > 3

How many total minutes did you hand express today?

☐ < 5 ☐ 5 – 10 ☐ 10 – 15 ☐ 15 – 20 ☐ 20 – 25 ☐ 25 – 30 ☐ > 30

How many milliliters of milk did you hand express today?

___________ mL

How confident do you feel with hand expression today?

☐ very uncomfortable ☐ uncomfortable ☐ neutral ☐ comfortable ☐ very comfortable

Did you feel any contractions today?

☐ yes ☐ no

If yes,

How many contractions did you feel every minute?

___________ / min

How often were these contractions occurring?

every ___________ hour(s)

Did you feel any discomfort today?

☐ yes ☐ no

If yes, please describe_______________________

____

____

My thoughts about hand expression today: ____

____

____

____

____
