# Supplementary material for: Teaching antenatal hand expression: a feasibility study in an inner urban U.S. hospital
Source: Int Breastfeed J. 2023 Aug 10;18:39. doi: 10.1186/s13006-023-00578-w (PMC10416364; doi:10.1186/s13006-023-00578-w)
Supplement: Supplementary file 2 — Additional file 2: Postpartum Survey. Assessment of participants in the hospital after delivery regarding satisfaction with hand expression and issues encountered, breastfeeding plans and barriers, and exclusive breastfeeding practices [file 13006_2023_578_MOESM2_ESM.pdf]

Additional File 2: Postpartum survey

Post Delivery Survey Participant ID________________ Date__________________

1. **Have you initiated breastfeeding?** Yes No

2. **Have you ever breastfed in the past?** Yes No

3. **Have you ever done hand expression before this study?** Yes No

4. **Have you practiced hand milk expression at home?** Yes No
   If yes, how many times have you practiced at home? ____________

5. **Did you encounter any problems when you practiced?** Yes No
   If yes, what kind of problems have you encountered?

6. **How satisfied were you with the experience of hand milk expression?**

   1 2 3 4 5 6 7 8 9 10

   Not satisfied at all Extremely satisfied

7. **How helpful was the hand expression education provided by the research team?**

   1 2 3 4 5 6 7 8 9 10

   Not helpful at all Extremely helpful

8. **Do you think practicing hand expression helped initiating breastfeeding easier?**

   1 2 3 4 5 6 7 8 9 10

   Not at all Extremely so

9. **Do you plan on exclusively breastfeeding?** Yes No
   If yes, how likely will you continue exclusively breastfeeding?

   1 2 3 4 5 6 7 8 9 10

   Not likely at all Extremely likely

   If no or not likely to continue, name any barriers preventing you from exclusively breastfeeding.

10. **How likely would you recommend hand milk expression to your friends and families?**

    1 2 3 4 5 6 7 8 9 10

    Not likely at all Extremely likely
